# sFgl2-Treg Positive Feedback Pathway Protects against Atherosclerosis

**DOI:** 10.3390/ijms24032338

**Published:** 2023-01-25

**Authors:** Tianhui An, Mengyuan Guo, Cheng Fan, Shiyuan Huang, Hui Liu, Kun Liu, Zhaohui Wang

**Affiliations:** 1 Department of Geriatrics, Union Hospital, Tongji Medical College, Huazhong University of Science and Technology, Wuhan 430022, China; 2 Department of Cardiology, Union Hospital, Tongji Medical College, Huazhong University of Science and Technology, Wuhan 430022, China; 3 Department of Cardiology, Boston Children’s Hospital, Harvard Medical School, Boston, MA 02114, USA

**Keywords:** atherosclerosis, Treg cells, sFgl2, Smad2, Smad3

## Abstract

Soluble fibrinogen-like protein 2 (sFgl2), a novel effector of regulatory T cells (Tregs), has been demonstrated to have potent immunosuppressive functions. Multiple studies indicate that Tregs could exert important atheroprotective effects, but their numbers gradually decrease during atherogenesis. The receptor of sFgl2 can be expressed on Treg precursor cells, while the role of sFgl2 on Treg differentiation and atherosclerosis progression remains unclear. Firstly, we detected that the sFgl2 was decreased in humans and mice with atherosclerotic diseases and was especially lower in their vulnerable plaques. Then, we used both Adeno-associated virus-sFgl2 (AAV-sFgl2)-injected ApoE^-/-^ mice, which is systemic overexpression of sFgl2, and sFgl2^Tg^ApoE^-/-^ bone marrow cells (BMC)-transplanted ApoE^-/-^ mice, which is almost immune-system-specific overexpression of sFgl2, to explore the role of sFgl2 in atherosclerosis. Our experiment data showed that AAV-sFgl2 and BMT-sFgl2 could reduce atherosclerotic area and enhance plaque stability. Mechanistically, sFgl2 increases the abundance and immunosuppressive function of Tregs, which is partly mediated by binding to FcγRIIB receptors and phosphorylating Smad2/3. Collectively, sFgl2 has an atheroprotective effect that is mainly achieved by forming a positive feedback pathway with Treg. sFgl2 and Treg could synergistically protect against atherosclerosis.

## 1. Introduction

Atherosclerosis (AS) is the leading cause of cardiovascular and cerebrovascular diseases, accounting for almost one in four deaths worldwide [1,2]. The pro-inflammatory damage/anti-inflammatory repair effects mediated by inflammatory cells and their subtypes in atherosclerotic plaques play a key role in regulating the progress of disease [3,4].

Studies have found that during the development of AS, lipoproteins enter the subintima of arteries and are presented to the naïve T cells in peripheral blood, lymph nodes, spleen, or other immune organs by antigen-presenting cells [5,6,7]. The naïve T cells then proliferate, infiltrate into plaques, and further differentiate into regulatory T (Treg) or effector T (Teff) cells [5]. Treg cells can mediate powerful anti-inflammatory and tissue repair effects and are mainly distributed in relatively stable areas of plaque. Multiple studies have indicated that Tregs could secrete IL-10 and TGF-β to reduce intraplaque inflammation, promote the conversion of pro-inflammatory M1 cells to anti-inflammatory M2 cells, and increase the deposition of stiffer type I collagen fibers to induce tissue repair, thus enhancing plaque stability and delaying plaque progression [8,9]. Conversely, Teff has a pro-inflammatory effect and could promote plaque instability [10].

Tregs have potent protective effects in AS, and adoptive transfer of Tregs has become a feasible anti-inflammatory treatment strategy. However, adoptive transfer of allogeneic Tregs has adverse effects such as clearance by the graft-versus-host reaction; polyclonal autologous Tregs also have disadvantages such as difficulty in obtaining sufficient cells, unsustainable anti-inflammatory effects, and changes in functional status during purification and transfer [8,11,12]. How to effectively upregulate Tregs has become an urgent problem to be solved in AS.

sFgl2 is a novel effector of Tregs and is determined to have powerful immunosuppressive activity in viral hepatitis, autoimmune nephropathy, and heart transplantation [13,14,15]. Unlike membrane-associated Fgl2 (mFgl2), which functions as a prothrombinase, sFgl2 exerts its anti-inflammatory effects by binding to inhibitory FcγRIIB receptors on T cells, dendritic cells, and other antigen-presenting cells [13,16,17]. One of our previous studies revealed that in patients with acute coronary syndrome (ACS), the plasma sFgl2 was decreased, which was in line with lowered Tregs frequency and inversely correlated with clinical outcome [18]. In addition, based on our analysis of relevant studies in the GEO database, we found that the expression of the sFgl2 gene was reduced in human arterial plaques and lower in unstable ones, suggesting that sFgl2 may play a role in stabilizing plaques and delaying atherosclerosis progression, but the exact mechanism remains unknown. Furtherly, there is research showing that the number and immunosuppressive function of Foxp3^+^ Treg cells in sFgl2-overexpressing heart transplant recipients were higher than those in the control group and that the Treg/Teff balance could be skewed to a Treg-dominated anti-inflammatory state [19]. However, whether sFgl2 can promote the differentiation of Treg cells in atherosclerosis has not been reported.

Collectively, considering that sFgl2 is a novel and potentially significant protein in atherosclerosis and the versatile contribution of Tregs in modulating atherosclerotic immunity, we examined the function and underlying pathological mechanisms of sFgl2 in both human atherosclerosis and BMT/AAV-sFgl2-injected atherosclerotic mice. The results revealed that sFgl2 was decreased in patients with acute stroke and atherosclerotic mice, and sFgl2 could attenuate atherosclerosis by increasing the abundance and immunosuppressive function of Tregs. Mechanistically, sFgl2 increases the abundance and immunosuppressive function of Tregs partly through binding to FcγRIIB receptors and phosphorylating Smad2/3.

## 2. Results

### 2.1. sFgl2 Was Decreased in Humans and Mice with Atherosclerotic Disease and Negatively Correlated with the Plaque Stability

**Figure 1:**
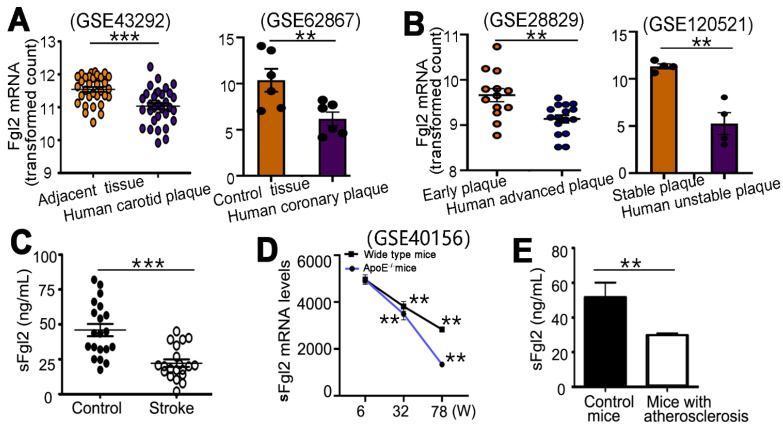
sFgl2 decreased in both human and mouse atherosclerotic diseases. (**A**) Quantification of sFgl2 mRNA relative expression between human carotid plaques and adjacent carotid tissues from microarray data (GSE43292) and between human coronary plaques and control tissues (GSE62867). (**B**) Quantification of sFgl2 mRNA relative expression between advanced human plaques and early plaques (GSE28829) and between human unstable plaques and stable plaques (GSE120521). (**C**) Elisa assay detected changes of sFgl2 protein levels in the plasma of healthy controls (N = 20) and acute stroke patients (N = 20). (**D**) In the aorta of wide-type or ApoE^-/-^ mice fed a Western diet, sFgl2 gradually decreased with time. (**E**) sFgl2 decreased in atherosclerotic mice compared with controls. ** *p* < 0.01, *** *p* < 0.001. Data were represented as mean ± SEM.

We analyzed human microarray data (GEO GSE43292) and found the sFgl2 mRNA expression was significantly lower in human carotid plaques (11.03 ± 0.09) than that in adjacent carotid tissues (11.54 ± 0.07, *p* < 0.001), (Figure 1A). Then, the microarray data (GEO GSE62867) showed that sFgl2 in human coronary plaque (6.19 ± 0.72) was also significantly lower than that in control tissues (10.36 ± 1.24, *p* < 0.005) (Figure 1A). Furthermore, the sFgl2 mRNA in human advanced plaques (9.14 ± 0.83) was lower than that in early plaques (9.66 ± 1.46, *p* < 0.005), as shown in microarray data (GEO GSE28829) (Figure 1B). In addition, the microarray data (GEO GSE120521) showed that sFgl2 in human unstable plaque (52.5 ± 11.6) was also significantly lower than that in stable ones (113.2 ± 2.7, *p* < 0.005) (Figure 1B). These results revealed that the mRNA of sFgl2 is decreased in atherosclerotic plaques and is lower in unstable plaques.

To explore the changes of sFgl2 protein in human atherosclerotic disease, we investigated its level in patients with acute ischemic stroke and found that plasma sFgl2 in acute stroke patients (22.2 ± 2.6 ng/mL) was significantly lower than that in healthy volunteers (45.9 ± 4.3 ng/mL, *p* < 0.001), which was consistent with our previous findings in patients with the acute coronary syndrome (ACS) (Figure 1C).

In mice, we obtained consistent findings. After analyzing microarray data (GEO GSE40156), we detected the sFgl2 mRNA expression was gradually decreased with the increase of a week both in the aorta of wild-type and ApoE^-/-^ mice fed a Western diet (Figure 1D). Furtherly, our results showed that the protein levels of plasma sFgl2 in atherosclerotic mice (29.5 ± 1.3 ng/mL) were significantly lower than that in non-atherosclerotic mice (51.4 ± 8.7 ng/mL, *p* < 0.005) (Figure 1E).

### 2.2. sFgl2-AAV Reduced Atherosclerotic Lesion and Stabilized Plaque in ApoE^−/−^ Mice

**Figure 2:**
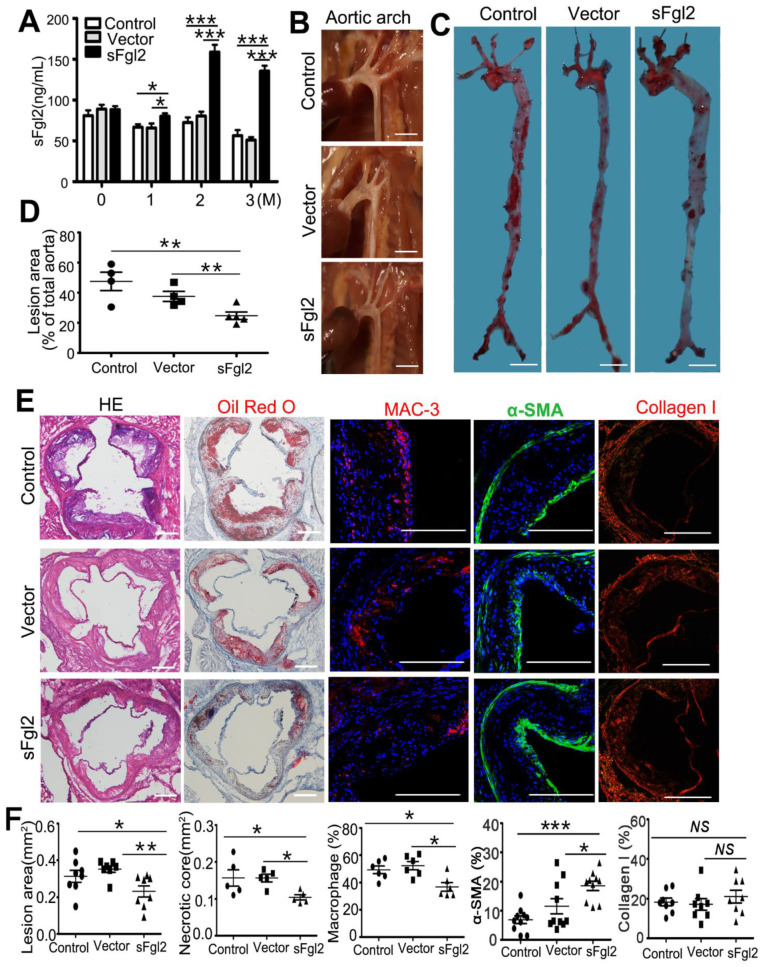
sFgl2-AAV alleviates artery burden and protects against atherosclerosis. (**A**) The expression levels of sFgl2 in PBS-, Vector-AAV-, and sFgl2-AAV- tail-vein-injected ApoE^-/-^ mice were detected by Elisa after feeding Western diet for 0, 1, 2, and 3 M. (**B**) Aortic arch lesion area in the three groups. Scale bar = 2 mm. (**C**) Oil Red O staining of the aorta en face lesion area. Scale bar = 500 μm. (**D**) Quantification of relative lesions area of the aorta. (**E**) Representative photomicrographs and (**F**) quantifications result of HE, Oil Red O, MAC-3, α-SMA, and collagen I. Scale bar = 200 μm. NS = Not significant, * *p* < 0.05, ** *p* < 0.01, *** *p* < 0.001. Data were represented as mean ± SEM.

Plasma sFgl2 was evaluated by ELISA after 0, 1, 2, and 3 months of PBS, Vector-AAV, or sFgl2-AAV injected into the tail veins of ApoE^−/−^ mice. The results showed sFgl2-AAV could stably upregulate the expression of sFgl2 compared with controls after 1, 2, and 3 months of virus or PBS injection (Figure 2A).

sFgl2-AAV could protect against atherosclerosis. Morphometric studies showed that in sFgl2-AAV-injected mice, the plaque area of aortic arch was smaller, and Oil O stained-lesion area of *en face* aorta decreased by 13% (from 37.6 ± 3.3 to 24.7± 2.5%) compared to the vector group (Figure 2B–D). Furthermore, sFgl2-AAV decreased the aortic root lesion area (H&E), necrotic core area (Oil Red O), and macrophages (MAC-3) from 0.35 ± 0.02 to 0.23 ± 0.03 mm^2^, 0.16 ± 0.01 to 0.10 ± 0.00 mm^2^, and 52.3 ± 3.1 to 36.8 ± 3.1% mm^2^, respectively (Figure 2E,F). In addition, the smooth muscle cell-positive areas (α-SMA) were more increased in the sFgl2-AAV group than in the vector group, but there was no difference in the collagenI area (Sirius Red Staining) between groups (Figure 2E,F). Additionally, sFgl2-AAV had no impact on the numbers of peripheral blood neutrophils as compared to controls (Supplementary Figure S1).

### 2.3. sFgl2-AAV Increased the Abundance and Function of Tregs by Binding to FcγRIIB Receptors and Phosphorylating Smad2/3

**Figure 3:**
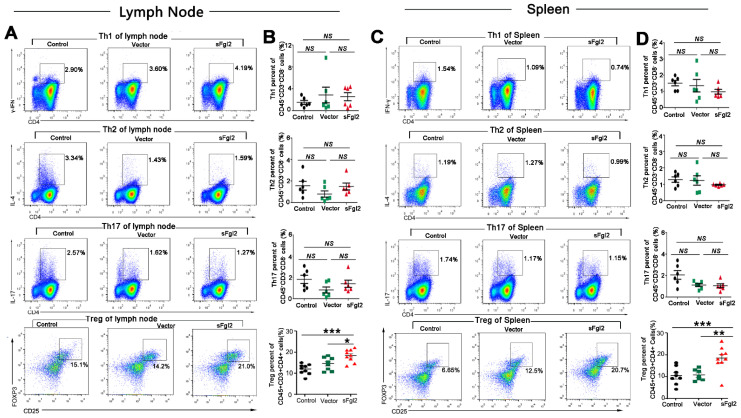
sFgl2-AAV increased the proportion of Tregs and had no impact on Th1, Th2, and Th17 cells’ percentage in the lymph nodes and spleen of atherosclerotic mice. (**A**) Representative flow cytometry analysis of Th1 (CD45^+^CD3^+^CD8^-^CD4^+^IFN-γ^+^), Th2 (CD45^+^CD3^+^CD8^-^CD4^+^IL-4^+^), Th17 (CD45^+^CD3^+^CD8^-^CD4^+^IL-17^+^), and Treg (CD45^+^CD3^+^CD4^+^ CD25^+^Foxp3^+^) cells in spleen of PBS-, Vector-AAV-, and sFgl2-AAV- tail-vein-injected ApoE^-/-^ mice. Left plots show gating of IFN-γ^+^, IL-4^+^, IL-17^+^, and Foxp3 by FCM. (**B**) Quantification results of (**A**). (**C**,**D**) Representative flow cytometry analysis and quantification results of Th1, Th2, Th17, and Treg cells in lymph nodes of the above mice. NS = Not significant, * *p* < 0.05, ** *p* < 0.01, *** *p* < 0.001. Data were represented as mean ± SEM.

**Figure 4:**
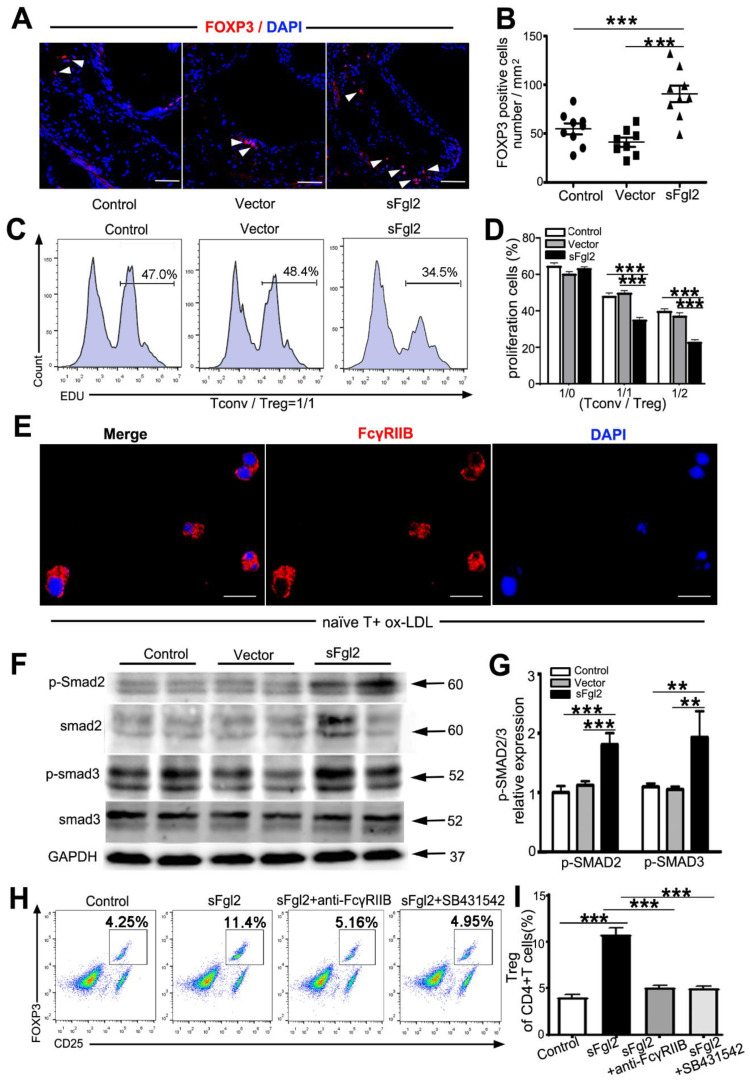
(**A**) Immunofluorescent staining and (**B**) quantification of Treg cells (Foxp3^+^) in the aortic root cross-sections of PBS-, Vector-AAV-, and sFgl2-AAV- tail-vein-injected ApoE^-/-^ mice. Scale bar = 100 μm. (**C**) Firstly, CD4+CD25- (T_Conv_) cells from WT mice and CD4+CD25+ (Treg) cells from the above three groups of ApoE^-/-^ mice were sorted by FACS. Then, the EDU-labeled T_Conv_ cells were co-cultured with Treg cells at ratios of 1/0,1/1, and 1/2 on CD3/CD28 pre-incubated plates. Representative histograms for T_Conv_ proliferation were shown. (**D**) Bars represent the percentage of proliferating T_Conv_ cells (N = 3). (**E**) Naïve T cells were obtained from atherosclerotic ApoE^-/-^ mice by FACS, and the expression of FcγRIIB receptors was detected after stimulation of these cells with 50 ug/mL ox-LDL for 24 h. Scale bar= 10 μm. (**F**) Western blotting analysis of Smad2/3 and *p*-Smad2/3 expression in the naïve T cells of the above mice. (**G**) *p*-Smad2 or *p*-Smad3 was normalized to total protein levels, (N = 4). (**H**) Representative flow cytometry analysis of Treg cells (CD4^+^CD25^+^Foxp3^+^) in the PBS, sFgl2, sFgl2 +anti-FcγRIIB, or sFgl2 +SB431542-treated para-aortic lymph node cells in ApoE^-/-^ mice. Numbers represent the percentage of Treg cells in CD45^+^CD3^+^CD4^+^ T cells. (**I**) Quantification results of (**H**). ** *p* < 0.01, *** *p* < 0.001. Data were represented as mean ± SEM.

We analyzed the effect of sFgl2-AAV on the proportion of Th1 (CD45^+^ CD3^+^ CD4^+^ CD8^-^ IFN-γ^+^), Th2 (CD45^+^ CD3^+^ CD4^+^ CD8^-^ IL4^+^), Th17 (CD45^+^ CD3^+^ CD4^+^ CD8^-^ IL17^+^), and Treg (CD45^+^ CD3^+^ CD4^+^ CD25^+^ Foxp3^+^) cells in lymph nodes and spleen by flow cytometry. Our data revealed, compared with Vector-AAV, that sFgl2-AAV exerted no influence on the frequency of lymph node Th1 (2.56 ± 0.75 vs. 2.86 ± 1.44%), Th2 (1.49 ± 0.32 vs. 0.79 ± 0.30%), Th17 (1.40 ± 0.34 vs. 0.82 ± 0.28%), and splenic T helper cells including Th1 (0.98 ± 0.15 vs. 1.35 ± 0.39%), Th2 (0.96 ± 0.03 vs. 1.23 ± 0.29%), and Th17 (1.05 ± 0.18 vs. 1.11 ± 0.14%) cells (Figure 3A–D). However, sFgl2 could surprisingly increase the proportion of lymph node Tregs (18.3 ± 1.0 vs. 14.5 ± 1.1%) and splenic Tregs (18.5 ± 1.8 vs. 10.8 ± 0.9%), respectively (Figure 3A–D). Immunofluorescence staining of the aortic root plaque further showed that sFgl2-AAV increased Foxp3^+^Tregs (54.8 ± 5.6 vs. 90.7 ± 8.4/mm^2^, Figure 4A,B). Since sFgl2 is mainly secreted by Tregs, here we demonstrate that there exists a positive feedback pathway between sFgl2 and Tregs in atherosclerotic mice.

In order to investigate whether sFgl2 could affect the Tregs’ suppressive function, we sorted CD4^+^C25^-^ Tconv cells from WT mice and CD4^+^C25^+^ Treg cells from PBS-, Vector-AAV-, and sFgl2-AAV-injected mice by FACS. Then, we co-cultured EDU-labeled Tconv cells with Tregs at ratios of 1:0, 1:1, and 1:2 at CD3/CD28 pre-incubated 12-well-round bottom plates for 72 h. Flow cytometry results showed that Tregs in the sFgl2-AAV group could inhibit the percentages of Tconv proliferation more effectively than in control groups (Tconv/Tregs = 1:0, control vs. vector vs. sFgl2 = 65.10 ± 1.20 vs. 60.37 ± 1.18 vs. 63.47 ± 0.97; Tconv/Tregs = 1:1, control vs. vector vs. sFgl2 = 48.45 ± 1.27 vs. 49.93 ± 1.43 vs. 35.23 ± 1.10; Tconv/Tregs = 1:2, control vs. vector vs. sFgl2 = 40.17 ± 1.28 vs. 37.50 ± 1.73 vs. 23.27 ± 1.11) at different Tconv/Treg ratios (Figure 4C, D). Then, we explored the possible pathways of how sFgl2 increased the abundance of Tregs. As we have described, Treg/Teff cells are differentiated from naïve T cells in response to different stimulating factors, so we first tested whether naïve T cells express the sFgl2′s receptor FcγRIIB. Naïve T cells were obtained from atherosclerotic ApoE^-/-^ mice by flow cytometry sorting, and the expression of FcγRIIB receptors was detected after stimulation of these cells with 50 ug/mL ox-LDL for 24 h. The results of immunofluorescence staining confirmed that naïve T cells could express FcγRIIB receptors (Figure 4E).

As reported, Smad2 cooperated with the Smad3 protein, which could bind to the CNS1 enhancer region or form an enhanceosome complex along with others at the promoter of Foxp3 to promote TGF-β-mediated Tregs induction. Here, we investigated whether sFgl2 mediated Tregs differentiation via phosphorylation of Smad2/3. Western blot analysis detected that the relative expression levels of *p*-Smad2 (1.81 ± 0.19 vs. 1.00 ± 0.11) and *p*-Smad3 (1.93 ± 0.44 vs. 1.10 ± 0.05) were increased in the sFgl2-AAV group compared with the control, while there was no difference in Smad2 and Smad3 relative expression among groups (Figure 4F,G).

Next, we used anti-FcγRIIB and SB431542 (blocker of *p*-Smad2/3) to study the potential role of FcγRIIB and p-Smad2/3 in the differentiation of Tregs. In vitro experiments showed that compared with the control (4.00 + 0.32%), sFgl2 (10.76 + 0.69%) can effectively promote the differentiation of naïve T to Treg cells, while sFgl2+ anti-FcγRIIB (5.11 + 0.16%) and sFgl2+ SB431542 (4.96 + 0.25%) could obviously block the above process (Figure 4H,I).

Taken together, sFgl2 could increase the abundance and function of Tregs by binding to FcγRIIB receptors and phosphorylating Smad2/3.

### 2.4. Bone-Marrow-Derived sFgl2 Could Affect Cellular Plaque Composition and Attenuate Atherosclerosis in Mice

**Figure 5:**
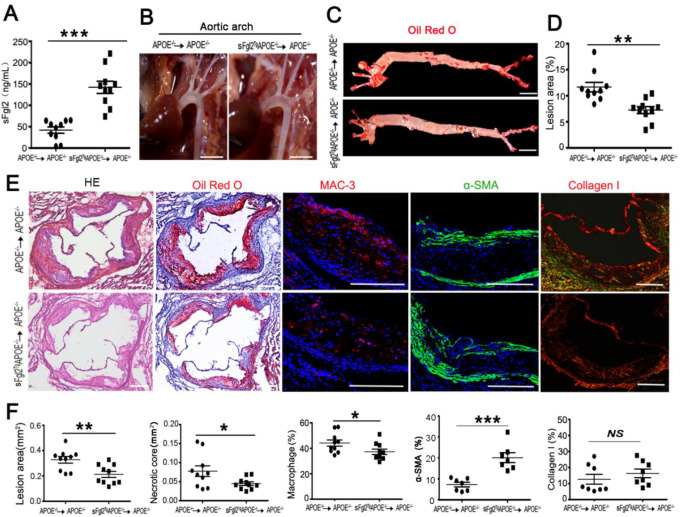
Over-expression of bone-marrow-derived sFgl2 could affect cellular plaque composition and attenuate atherosclerosis in mice. (**A**) The expression level of sFgl2 in irradiated ApoE^-/-^ mice reconstituted with sFgl2^Tg^ApoE^-/-^ or ApoE^-/-^ bone marrow was detected by Elisa. (**B**) Aortic arch lesion area in two groups. Scale bar = 2 mm. (**C**) Oil Red O staining of the aorta en face lesion area. Scale bar = 5 mm. (**D**) Quantification of relative lesions area of full-length aorta. (**E**) Representative photomicrographs showing HE, Oil Red O, MAC 3, α-SMA, and collagen I stained area in cross-sections of the aortic root. Scale bar = 200 μm. (**F**) Quantifications results of HE, Oil Red O, Mac-3, α-SMA, and collagenI. NS = Not significant, * *p* < 0.05, ** *p* < 0.01, *** *p* < 0.001. Data were represented as mean ± SEM.

Unlike systemic overexpression of sFgl2 by tail vein injection of AAV- sFgl2, we developed a model of immune-system-specific overexpression of sFgl2 by using sFgl2 transgenic mice in combination with bone marrow transplantation technology. First, we generated and propagated homozygous sFgl2^Tg^ApoE^-/-^ or ApoE^-/-^ mice; second, we irradiated ApoE^-/-^ mice and transplanted them with bone marrow from sFgl2^Tg^ApoE^-/-^ or ApoE^-/-^ mice; at last, after 4 weeks of transition, all recipient mice were fed a Western diet for 12 weeks. The plasma sFgl2 in two groups were measured before they were fed a Western diet, and the results showed that sFgl2 levels in sFgl2^Tg^ApoE^-/-^ chimeras (142.3 ± 14.3 ng/mL) were significantly higher than that in the control group (41.9 +7.4 ng/mL) (Figure 5A). Then, lesion size in the aortic arch and full length of the aorta were analyzed. The results showed bone-marrow-derived sFgl2 could reduce the plaque area in the aortic arch (Figure 5B). *En face* Oil Red O staining further revealed the relative plaque burden was less in sFgl2^Tg^ApoE^-/-^ chimeras (7.2 ± 0.76%) than that in ApoE^-/-^ chimeras (11.6 ± 0.92%, Figure 5C,D). Further analysis of aortic sinus plaque compositions revealed that sFgl2 decreased the lesion area (H&E), necrotic core area (Oil Red O), and macrophages (MAC-3) from 0.33 ± 0.03 to 0.21 ± 0.03 mm^2^, 0.08 ± 0.01 to 0.04 ± 0.00 mm^2^, and 50 ± 4.8 to 36 ± 2.6%, respectively (Figure 5E,F). Immunofluorescence staining of α-SMA and Sirius red staining indicated that sFgl2 could increase smooth muscle cell percentages and have no influence on collagen I area compared with controls (Figure 5E,F). These results revealed that bone-marrow-derived sFgl2 attenuated atherosclerosis in mice.

### 2.5. Bone-Marrow-Derived sFgl2 Restored the Numbers and Function of Treg Cells by Binding to FcγRIIB Receptors and Phosphorylating Smad2/3

**Figure 6:**
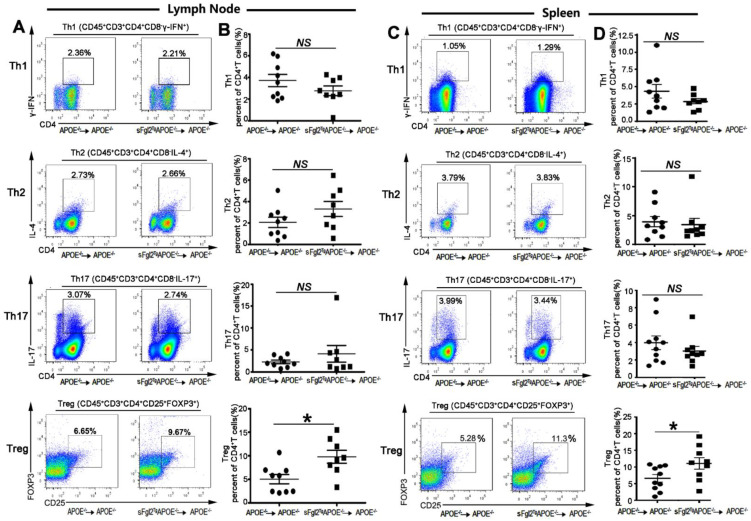
Over-expression of bone-marrow-derived sFgl2 increased Treg differentiation without affecting the population of T helper cells. (**A**) Representative flow cytometry analysis of Th1 (CD45^+^CD3^+^CD8^-^CD4^+^IFN-γ^+^), Th2 (CD45^+^CD3^+^CD8^-^CD4^+^IL-4^+^), Th17 (CD45^+^CD3^+^CD8^-^CD4^+^IL-17^+^), and Treg (CD45^+^CD3^+^CD4^+^CD25^+^Foxp3^+^) cells’ percent among CD4^+^ T cells in spleen of irradiated ApoE^-/-^ mice reconstituted with sFgl2^Tg^ApoE^-/-^ or ApoE^-/-^ bone marrow. Numbers represented the percentage of Th1, Th2, Th17, and Treg cells in CD4^+^T cells. (**B**) Quantification results of (**A**). (**C**) FCM analysis of Th1, Th2, Th17, and Treg cells percent among CD4^+^ T cells in lymph nodes of irradiated ApoE^-/-^ mice reconstituted with sFgl2^Tg^ApoE^-/-^ or ApoE^-/-^ bone marrow. (**D**) Quantification results of (**C**). NS = Not significant, * *p* < 0.05. Data were represented as mean ± SEM.

**Figure 7:**
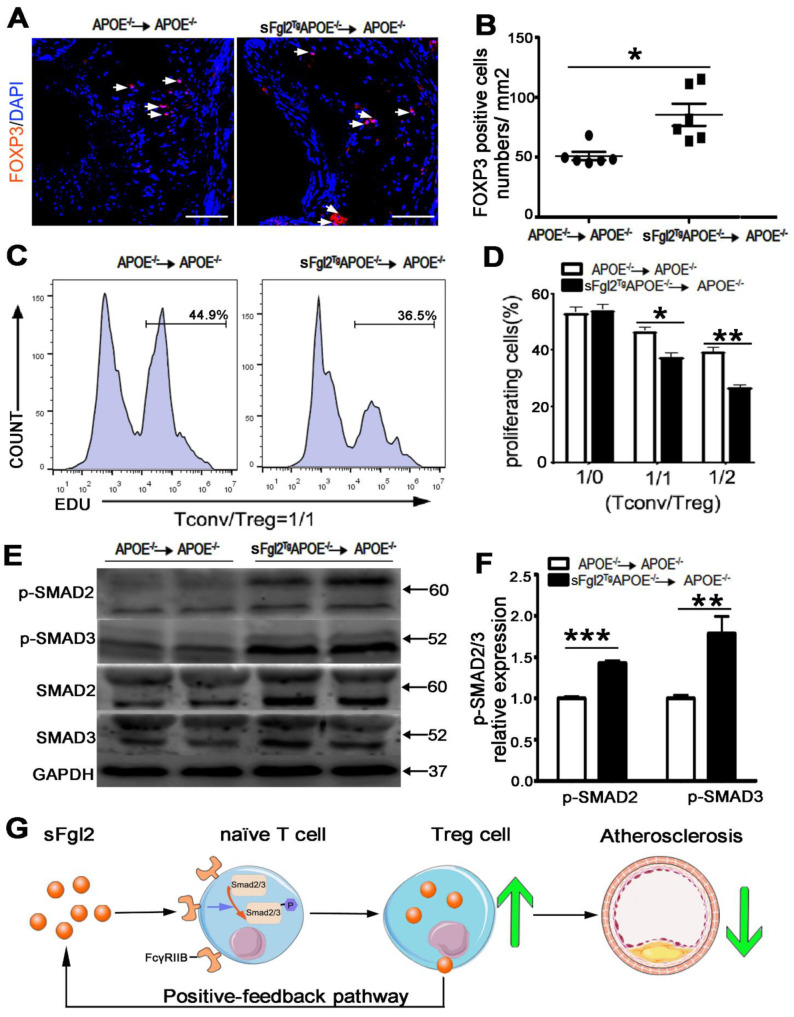
Bone marrow-derived sFgl2 increased the Tregs population and immunosuppressive function by binding to FcγRIIB receptors and phosphorylating Smad2/3. (**A**) Immunofluorescent staining and (**B**) quantification of Foxp3 in the aortic root cross-sections of the irradiated ApoE^-/-^ mice reconstituted with sFgl2^Tg^ApoE^-/-^ or ApoE^-/-^ bone marrow. Triangles indicate positive stained cells. Scale bar = 100 μm. (**C**) Firstly, CD4+CD25- (T_Conv_) cells from WT mice and CD4+CD25+ (Treg) cells from two groups of irradiated mice were sorted by FACS. Then, the EDU-labeled T_Conv_ cells were co-cultured with Treg cells at ratios of 1/0,1/1, and 1/2 on CD3/CD28 pre-incubated plates. Representative histograms for T_Conv_ proliferation were shown. (**D**) Bars represent the percentage of proliferating T_Conv_ cells, (N = 3). (**E**) Representative Western blot gel depicts the protein expression of phosphorylated SMAD2/3 (p-SMAD2/3) and total levels of SMAD2/3 in the above mice. (**F**) The relative percent of p-Smad2/3 in total Smad2/3, (N = 4). (**G**) Schematic diagram depicting the positive-feedback pathway between sFgl2 and Treg to protect against atherosclerosis. * *p* < 0.05, ** *p* < 0.01, *** *p* < 0.001. Data were represented as mean ± SEM.

Here, we analyzed whether the bone-marrow-derived sFgl2 could change proportions of Th1 (CD45^+^CD3^+^CD4^+^CD8^-^IFN-γ^+^), Th2 (CD45^+^CD3^+^CD4^+^CD8^-^IL4^+^), Th17 (CD45^+^CD3^+^CD4^+^CD8^-^IL17^+^), and Treg (CD45^+^CD3^+^CD4^+^CD25^+^Foxp3^+^) cells in spleen and lymph nodes by FCM. The results showed that sFgl2 exerted no influence on the percentages of Th1 (from 3.73 ± 0.50 to 2.77 ± 0.45%), Th2 (from 2.06 ± 0.49 to 3.31 ± 0.70%), and Th17 (from 2.26 ± 0.40 to 4.10 ± 0.91%) cells in lymph nodes (Figure 6A,B). In addition, sFgl2 could not affect Th1 (from 3.46 ± 0.79 to 2.30 ± 0.31%), Th2 (from 3.93 ± 0.90 to 3.42 ± 1.08%), and Th17 (from 3.98 ± 0.79 to 3.02 ± 0.54%) cells in splenic T helper cells (Figure 6C,D). Then, we found sFgl2 could increase the proportion of Tregs (from 5.37 ± 0.94 to 9.33 ± 1.30%) in lymph nodes (Figure 6A, B) and Tregs (from 7.11 ± 0.98 to 10.54 ± 0.98%) in spleens (Figure 6C,D). Additionally, bone-marrow-derived sFgl2 had no impact on the numbers of peripheral blood neutrophils as compared to controls (sup. Figure S2). Furthermore, we detected Tregs (Foxp3^+^ cells) in the aortic sinus of mice and found that sFgl2 could also increase Treg cell numbers in atherosclerotic plaques (from 60.0 ± 3.5 to 85.4 ± 9.2/mm^2^; Figure 7A,B).

To further investigate whether sFgl2 can affect the immunosuppressive function of Tregs, we sorted CD4^+^CD25^+^ Tregs from spleen of the sFgl2^Tg^ApoE^-/-^ or ApoE^-/-^ chimeras by FCM. Then, we co-cultured EDU-labeled CD4^+^CD25^-^ Tconv cells (sorted from WT mice) with Tregs at ratios of 1:0, 1:1, and 1:2 for 72 h on CD3/CD28 pre-incubated 12-well-round bottom plates as described before. Compared with the control, Tregs from sFgl2^Tg^ApoE^-/-^ chimeras suppressed the proliferation of Tconv at different Tconv/Treg ratios (Tconv/Tregs = 1:0, control vs. sFgl2 = 53.33 ± 1.98 vs. 54.27 ± 2.17; Tconv/Tregs = 1:1, control vs. sFgl2 = 48.87 ± 1.49 vs. 37.60 ± 1.46; Tconv/Tregs = 1:2, control vs. sFgl2 = 39.47 ± 1.58 vs. 26.70 ± 1.02) (Figure 7C,D).

In our study, sFgl2 increased the level of p-Smad2 (from 1.00 ± 0.02 to 1.43 ± 0.30) and p-Smad3 (from 1.00 ± 0.04 to 1.79 ± 0.21) but had no significant influence on Smad2 and Smad3 expression (Figure 7E,F), which suggested p-Smad2/3 may participate in the sFgl2-mediated Tregs restoration.

Finally, we depict our study in a diagram. sFgl2 is a novel secretory protein of Tregs, and it induces Smad2/3 phosphorylation by binding to the FcγRIIB receptor expressed on naïve T cells, resulting in the increased differentiation of Tregs. sFgl2 and Tregs form a positive feedback pathway, and they act synergistically to slow the progression of atherosclerosis (Figure 7G).

## 3. Discussion and Conclusions

We found that the sFgl2 mRNA was decreased in human and mice atherosclerotic plaques and lower in unstable ones by analyzing the related research in a public database, and then we detected that the sFgl2 protein was also decreased in the plasma of patients with acute stroke and atherosclerotic mice. In both AAV-sFgl2-injected ApoE^-/-^ mice, which is systemic overexpression of sFgl2, and sFgl2^Tg^ApoE^-/-^ bone-marrow-cells-transplanted ApoE^-/-^ mice, which is almost immune-system-specific overexpression of sFgl2, we detected that sFgl2 reduced plaque area and enhanced plaque stability mainly by forming a positive feedback pathway with Treg cells. In addition, to the best of our knowledge, no studies have reported the above pathway in atherosclerosis. Mechanistically, sFgl2 increased the abundance and immunosuppressive function of Tregs partly through binding to FcγRIIB receptors and phosphorylating Smad2/3.

It is well known that sFgl2 is mainly secreted by Treg cells [20], and our experiments showed that sFgl2 promotes the upregulation of Tregs abundance and immunosuppressive function in both AAV-sFgl2-injected and BMT-sFgl2-transplanted mice. This positive feedback pathway is of great significance because Tregs have a strong protective role in atherosclerosis, but they progressively decrease during the development of atherosclerosis [21]. Adoptive transfer of Tregs is an effective strategy to delay the progression of atherosclerosis, but both allogeneic and autologous transplantation of Treg face a series of problems that limit their application, and the sFgl2-Treg pathway provides a possible solution to this issue [22,23].

In atherosclerosis, there are various approaches to upregulate Tregs, such as Treg supplementation, transgenic mice, or thymidine supplementation, but the disadvantages of these approaches are short-lived effects, inhibition of secretion of the mice’s own Treg cells or high costs, which largely limit their future clinical applications [8]. In contrast, sFgl2 can form a positive feedback pathway with Tregs, effectively upregulating Tregs and exerting a powerful protective effect against AS, which has promising clinical applications in the future.

The stability of Foxp3 determines Treg function. In our experiments, we believe the induced Tregs are able to stably express Foxp3 for the following reasons. First, we constructed atherosclerosis models with both AAV-sFgl2 and BMT-sFgl2 mice fed a Western diet for three months. At the end of the experiment, we detected Foxp3-labeled Tregs in atherosclerotic plaques, spleen, and lymph nodes and found that they were significantly elevated compared with the controls. If the induced Tregs did not stably express Foxp3, the Foxp3 would gradually decrease and would not remain significantly elevated compared to the controls at the end of 3 months. Second, our experiments found that sFgl2 was consistently elevated in AAV-sFgl2 and BMT-sFgl2 atherosclerotic mice at different time points of modeling (Figure 2A and Figure 5A). sFgl2 was able to promote Smad2 and Smad3 phosphorylation, thereby promoting Foxp3^+^ Treg cell differentiation, so we believe that sFgl2 could consistently and stably promote the expression of Foxp3 in Tregs. Third, the Foxp3^+^Treg-sFgl2 positive feedback pathway found in our experiment also gives us reason to be more confident that Foxp3 can be consistently and stably induced by Treg/sFgl2.

Neutrophils are also important cells involved in the progression of atherosclerosis [24,25]. Since some studies have reported BMT could change the population of neutrophils [26], we detected the numbers of neutrophils in peripheral blood by flow cytometry to exclude its possible interfering effect and found no difference in neutrophils among subgroups of two models. Additionally, lipids also play an important role in the development of atherosclerosis, and no significant changes, including total cholesterol and triglycerides, were detected in our experiment across sFgl2 overexpression and control groups (Supplementary Table S1).

In our experiment, we chose AAV-sFgl2-injected mice to study the role of sFgl2 in atherosclerosis. Adeno-associated virus is one of the most promising gene vectors, and many AAV drugs have even been approved for clinical use. Our study selected the AAV-9 viral vector, which could highly express sFgl2 systemically, particularly in the immune system, and confirmed its significant role in attenuating atherosclerosis, thus laying an early foundation for future clinical applications of sFgl2-AAV in atherosclerotic disease.

We also explored the role of sFgl2 in the BMT mice model, in which the immune systems of irradiated ApoE^-/-^ mice (recipient) are destroyed and then reconstituted with the marrow of sFgl2^Tg^ApoE^-/-^ mice (donor), thus selectively upregulating the sFgl2 gene in the immune system of the recipient. Bone marrow transplantation is a widely used model in atherosclerosis, which can eliminate interference from other systems or tissues, make the source of sFgl2 more certain, and better illustrate the role of sFgl2 in atherosclerosis.

In previous studies, the expression of FcγRIIB in CD4 T cells during the resting or activated stages was a controversial topic. Whether FcγRIIB is expressed on CD4^+^ T cells is a crucial issue that determines whether sFgl2 can act directly on CD4^+^ T cells and promote their differentiation. Some researchers believe that FcγRIIB is not expressed on CD4^+^ T cells [27], but many studies in recent years have disproved this view. Holgado et al. demonstrated that resting CD4^+^ T cells can express cell surface FCΓRIIB, while activation of CD4^+^ T cells significantly increased FcγRIIB expression on the cell surface or intracellularly [28]. Chauhan and several other groups have also confirmed the presence of the FcγRIIB receptor on CD4^+^ T cells [29,30]. Moreover, FcγRIIB-mediated signaling can stimulate the activation and differentiation of human naïve CD4^+^ T cells [29,30]. Consistently, in our experiments, we confirmed that CD4^+^ T cells could express FcγRIIB under ox-LDL stimulation using immunofluorescence staining.

There are some limitations in our experiments. In exploring how sFgl2 promotes Tregs differentiation, we only detected the phosphorylation of smad2/3 and did not detect other molecules that may be involved for the following reasons. In response to inflammation and other stimuli (for example, sFgl2 in our experiment), naive T cells differentiate into Treg cells. Foxp3 is an important molecule expressed by Tregs and plays a key role in the immune function of Tregs [31,32]. Phosphorylation of Smad2 and Smad3 is critical for the induction and maintenance of Foxp3 protein, but the exact mechanism is controversial [33,34,35]. Specifically, Foxp3 gene expression is controlled by a core promoter and at least three enhancers (conserved non-coding sequences, CNS 1-3) that contain binding sites for Smad3, and although Smad2 lacks DNA-binding activity, it could help Smad3 to function [34]. However, it has been shown that the presence of the Smad-binding structural domain on the Foxp3 gene does not necessarily indicate that Smad transcription factors must regulate the transcription of Foxp3, and even without Smad-binding sites in gene promoters or regulatory elements, Smad transcription factors may still bind to target genes and regulate Foxp3 gene expression indirectly through interactions with partners [34,35,36]. In conclusion, it has been demonstrated that phosphorylation of Smad2/3 has an important role in Foxp3 induction and maintenance, but the specific mechanism still needs a lot of research to explore. Therefore, in exploring how sFgl2 promotes Tregs differentiation, we only examined sFgl2 binding to the FcγRIIB receptor expressed on naive T cells, phosphorylating Smad2/3, promoting Foxp3 expression, and promoting Tregs differentiation.

### Conclusions

In summary, our study demonstrated that sFgl2 was decreased in atherosclerosis and could protect against atherosclerosis through binding to FcγRIIB receptors and phosphorylating Smad2/3. Interestingly, we found a positive feedback pathway between sFgl2 and Tregs in atherosclerosis. At last, sFgl2 could be a promising biomarker and even drug in the prevention and treatment of cardiovascular and cerebrovascular diseases in the future, though much effort is still needed in experimental and clinical studies.

## 4. Materials and Methods

### 4.1. Ethics

All ApoE^-/-^ mice (male, 5–8 weeks old) of C57BL/6 background were purchased from the Animal Center of Peking University (Beijing, China), and all sFgl2^Tg^ApoE^-/-^ mice (male, 5–8 weeks old) of C57BL/6 background were purchased from Biocytogen Co., Ltd. (Beijing, China). Procedures in the experiment were in compliance with the requirements of the National Institutes of Health (NIH) guidelines. The animal study was approved by The Institutional Animal Care and Utilization Committee at Tongji Medical College of Huazhong University of Science and Technology (HUST), China (IACUC No.: 2136). Approval for the human study was obtained from the Ethics Committee of Tongji Medical College of HUST, China (IORG No.: IORG0003571). Written informed consent was obtained from the patients or appropriate surrogates.

### 4.2. Human and Mice Plasma Sample Collection

In the Union Hospital (Wuhan, China), twenty patients with acute ischemic stroke admitted within 7 d of symptom onset were recruited. All the patients were 40–70 years old and were confirmed to have carotid atherosclerotic plaque by ultrasound or magnetic resonance angiography (MRA). Patients with small-artery occlusion and cardioembolism were excluded according to TOAST classification. Additionally, participants with peripheral vascular disease and symptomatic coronary heart disease were also excluded. Twenty matched healthy volunteers (45–70 years old) were recruited as controls. They had a normal electrocardiogram and had no history of cardiovascular disease, cerebrovascular disease, and peripheral atherosclerotic disease. In addition, all the subjects in the two groups with a fever of unknown origin, rheumatoid valvular heart disease, renal failure, viral hepatitis, or receiving immunosuppressive drugs were excluded. In addition, there were no significant differences in baseline data such as age, gender, and previous diseases between the above two groups. Blood was collected from the patients using tubes containing ethylenediaminetetraacetic acid (EDTA) at the time of magnetic resonance imaging (MRI) and ultrasound.

Mice blood samples were also collected into EDTA-containing tubes from BMT-recipient mice (after BMT surgery and 3 months after feeding Western diet) and AAV-injected mice (0, 1, 2, and 3 months after feeding Western diet) via the saphenous vein puncture.

All blood samples were centrifuged (300× *g*, 10 min), and then the plasma in the upper layer was collected and stored at −80 °C.

### 4.3. Animal Establishment and Treatment

The atherosclerotic mice model was established as described previously [37]. All sFgl2^Tg^ApoE^-/-^ and ApoE^-/-^ mice were maintained in a specific pathogen-free facility with a 12 h light/dark cycle at 22–25 °C. The BMT mice were divided into two groups: recipient mice receiving bone marrow of ApoE^-/-^ (control) and sFgl2^Tg^ApoE^-/-^ (sFgl2). They were given a Western diet for 3 months. On the other hand, after being fed a week of rodent diet, ApoE^-/-^ mice were also given a Western diet and randomly divided into 3 AAV (Gikai Genetics, Shanghai, China)-injected groups: tail vein injection with PBS (control), Vector-AAV (vector), and sFgl2-AAV (sFgl2) mice. According to the instructions, the reagent (1 × 10^11^VG of Vector-AAV or sFgl2-AAV) was resuspended with 250 μL of normal saline and then injected into the mouse tail vein using an insulin syringe (1/2 mL).

### 4.4. Bone Marrow Transplantation

All ApoE^-/-^ and sFgl2^Tg^ApoE^-/-^ mice were housed in a climate-controlled, light-regulated facility with a 12:12 h light–dark cycle. At seven weeks of age, the femurs and tibias of ApoE^-/-^ mice and sFgl2^Tg^ApoE^-/-^ mice were isolated, and the bone marrow cavity was washed with normal saline. To obtain bone marrow cell suspension, total bone marrow cells were passed through a 40 μm cell strainer. On the other hand, 5-week-old male ApoE^-/-^ mice were fed with water-added antibiotics (roxithromycin and gentamicin) for 2 weeks for infection prevention. They were then exposed to total body irradiation (9Gy; two times 4.5Gy, 3 h apart) [38]. Within 6 h, bone marrow cells of ApoE^-/-^ or sFgl2^Tg^ApoE^-/-^ mice were randomly injected into irradiated recipients (1.5 × 10^7^ cells per mouse) through the tail vein. All BMT recipient mice were fed with sterile feed and water added with antibiotics for four weeks. Then, they were fed with a Western diet and normal water for another three months.

### 4.5. Enzyme-Linked Immunosorbent Assay (ELISA)

After the blood of humans and mice were collected, they were kept at room temperature for 2 h and rotated for 10 min at 4 °C to separate the plasma. Then, the levels of sFgl2 in them were determined by sFgl2 ELISA kits of humans (Biolegend, San Diego, CA, USA) and mice (Biolegend, San Diego, CA, USA), respectively, according to the manufacturer’s instructions. Two biological replicates were performed for ELISA analysis.

### 4.6. Tissue Preparation and Immunofluorescence Staining

The American Heart Association guidelines for experimental atherosclerosis studies were used in the histological analyses of this study [39]. The tissue preparation and immunofluorescence staining were performed as described previously [37], and detailed steps were as follows. Hearts and aortas were carefully separated and excised from mice sacrificed. Hearts were bisected at the left and right auricular appendage levels, and the base of the heart and aortic root were filled with OCT and then frozen at −80 °C. Cryostat sections (6 μm) were prepared by Microtome cryostat at −20 °C, and then sections were stored at −80 °C.

The aortas and some cryostat sections were stained with Oil Red O (ORO) to calculate the percentage of the dyed sections to the total aorta or cross-sectional area, and the stained aortas were imaged by EOS 550D Digital SLR Camera (Canon, Tokyo, Japan). In addition, the serial tissue sections were selected to be stained with hematoxylin and eosin (H&E) or Sirius red staining and photographed by polarization microscope for collagen type distinction and quantification. The lesion size and foam cell area were measured on ZEN software (Zeiss, Jena, Germany). Sirius red staining positive area was quantified with Image J software. Parameters were scored or quantified in at least three sections in the same location per mouse.

To detect macrophages, smooth muscle cells, and Treg cells in atherosclerotic plaque, sections were stained with primary antibodies against MAC-3 (dilution 1:100, Abcam, Cambridge, England), α-SMA (dilution 1:100, Abcam, Cambridge, England), and Foxp3 (dilution 1:100, Abcam, Cambridge, England), respectively. After incubating the primary antibody, the corresponding second antibody and DAPI continued to incubate. All images were obtained on a TCS SP5 multiphoton laser scanning confocal microscope (Nikon, Tokyo, Japan). The means were calculated from five randomly selected microscopic fields in the plaque, and positive cells were analyzed by two blinded investigators with ImageJ (NIH, Bethesda, MD, USA). Data were expressed as mean numbers of cells per square millimeter.

### 4.7. Flow Cytometry

Flow cytometry was performed as described previously [40], and details were as follows.

#### 4.7.1. Cell Preparation

After AAV-injected and BMT-transplanted mice were euthanized with 100% CO2, peripheral blood was drawn into EDTA-containing tubes via cardiac puncture by an insulin syringe. Spleens and lymph nodes were excised after the blood vessels were perfused with PBS. The spleens and lymph nodes were ground in HBSS with the end of a 1 mL syringe (BD Bioscience, Franklin Lakes, NJ, USA) at 4 °C and then filtered through a 40 μm nylon mesh. Peripheral blood cells and filtered cells were lysed using a red blood cell lysing buffer (Sigma-Aldrich, St. Louis, MO, USA). Then, the cell suspensions were overlaid on Ficoll density gradient (Sigma-Aldrich, St. Louis, MO, USA) and then centrifuged (400× *g*, 25 min) to collect the cells in the interface (further analysis for T helper and Treg cells) or directly centrifuged (further analysis for neutrophils). Before antibody incubation, total viable cell numbers were determined using Neubauer chamber (BD Bioscience, Franklin Lakes, NJ, USA) with Trypan blue (Sigma-Aldrich, St. Louis, MO, USA).

#### 4.7.2. Antibody Incubation

For the analysis of helper T cell subsets, the cell suspensions derived from spleens or lymph nodes were cultured with a cell stimulation cocktail (eBioscience, San Diego, CA, USA) on a 6-well plate (BD Biosciences, Franklin Lakes, NJ, USA) in the incubator. After 4 h, the cells were collected and washed and then first incubated with antibodies against CD45 (eBioscience, San Diego, CA, USA), CD3 (Biolegend, USA), CD4 (eBioscience, San Diego, CA, USA), and CD8 (eBioscience, San Diego, CA, USA) for 30 min at 4 °C. After being washed twice with PBS, the cells were fixed with 4% phosphate-buffered paraformaldehyde, permeabilized, and then stained with antibodies against IFN-γ (eBioscience, San Diego, CA, USA), IL-4 (eBioscience, San Diego, CA, USA), and IL-17 (eBioscience, San Diego, CA, USA) for 30 min at 4 °C to detect intracellular antigens.

For the analysis of Treg cells, firstly, the cell suspensions derived from spleens and lymph nodes were stained with cell surface markers: CD3 (eBioscience, San Diego, CA, USA), CD4 (eBioscience, San Diego, CA, USA), and CD25 (eBioscience, San Diego, CA, USA). Secondly, the cell suspensions were added to Foxp3 fixation/permeabilization working solution and incubated for 60 min at 4 °C. Thirdly, they were permeabilized twice. Finally, they were incubated with the antibody against Foxp3 (eBioscience, San Diego, CA, USA) for 30 min and washed.

For the analysis of neutrophils, cell suspensions derived from peripheral blood were incubated with antibodies against CD45 (eBioscience, San Diego, CA, USA), Ly-6G (Biolegend, San Diego, CA, USA), and CD11b (eBioscience, San Diego, CA, USA). All antibodies were incubated for 30 min at 4 °C and washed twice.

All the above cells were transferred to tubes, placed in a 4 °C refrigerator, and then analyzed by FACS Aria^™^ II Cytometer (BD Bioscience, Franklin Lakes, NJ, USA).

### 4.8. Block FcγRIIB Receptors and p-SMAD2/3 with anti-FcγRIIB and SB431542, Respectively

We obtained naive cells (CD4^+^CD62L^high^CD44^low^CD25^−)^ from the thymus of ApoE^-/-^ mice using fluorescence-activated cell sorting (FACS) and stimulated them with ox-LDL. Then, we treated the cells with PBS, sFgl2 (Yeasen, Guangzhou, China), sFgl2+ anti-FcγRIIB (BD Pharmingen, Franklin Lakes, NJ, USA), or sFgl2+SB431542 (Selleck, Houston, Tex, USA) for 24 h, respectively. Finally, we analyzed the proportion of Treg cells in each group by flow cytometry.

### 4.9. Treg Cells Suppression Assay

The sFgl2^Tg^ApoE^-/-^ and ApoE^-/-^ chimer mice were sacrificed and soaked in 75% alcohol for 30 min, and then all steps followed were carried out in Clean Bench. Spleens and lymph nodes were excised, and single-cell suspensions were obtained as described in the “Flow cytometry” section.

The single-cell suspensions were incubated with antibodies against CD4 (eBioscience, San Diego, CA, USA) and CD25 (eBioscience, San Diego, CA, USA) for 30 min at 4 °C and then washed and sorted by a FACS Aria^™^ II Cytometer (BD Bioscience, Franklin Lakes, NJ, USA) to obtain CD4^+^CD25^+^ and CD4^+^CD25^-^ cell suspensions. After CD4^+^CD25^-^ cell suspensions (1 × 10^6^ cells/well) were stained with EDU (eBioscience, San Diego, CA, USA), they were co-cultured with CD4^+^CD25^+^ cell suspensions at the ratios of 1:0, 1:1, and 1:2 on CD3/CD28 pre-incubated 12-well plate for 72 h. The EdU incorporation assay was performed as described previously [41].

Single fluorescence controls were used to draw positive gating lines. Data about stained cells and cell proliferation were analyzed with FlowJo 7.6.1 (TreeStar Inc., Palo Alto, CA, USA).

The relative quantification of gene expression was performed using the comparative Ct method and expressed as 2^−ΔΔCt^.

### 4.10. Western Blot Analysis

Western blot analysis was performed as described previously [42]. Proteins were extracted from naïve T cells, which were obtained by flow-sorting from atherosclerotic para-aortic lymph nodes. The total proteins were then separated by 10% SDS-polyacrylamide gel electrophoresis (PAGE) and transferred to a nitrocellulose membrane. The membranes were blocked with 5% skim milk for 2 h and then incubated with primary antibodies against SMAD2 (dilution 1:1000, Abcam, Cambridge, England), SMAD3 (dilution 1:1000, Abcam, Cambridge, England), p-SMAD2 (dilution 2:1000, Abcam, Cambridge, England), and p-SMAD3 (dilution 2:1000, Abcam, Cambridge, England) overnight at 4 °C. Finally, they were incubated with HRP-conjugated secondary antibody (dilution 1: 2000, Biosci Biotech Co., Ltd., Wuhan, China) for 2 h. All samples were run-in duplicates. Target and GAPDH strips were visualized with ECL reagent (Pierce, Waltham, Mass, USA) on an autoradiography film (UVP OptiCam600, Upland, CA, USA).

### 4.11. Statistical Analysis

All experiments were randomized and blinded. Data were presented as mean ± SEM. Comparisons between 2 groups were calculated by 2-tailed Student t-tests (the paired t-test was used in Figure 1A and Figure 1B-right, and the independent t-test was used for all the rest). Multiple group comparisons were analyzed by ANOVA analysis with Bonferroni post hoc correction. SPSS 21.0 (SPSS Inc., Armonk, NY, USA) and GraphPad Prism 7.0 were used for all these analyses. A two-sided *p* < 0.05 was considered statistically significant.

## Data Availability

The data that support the findings of this study are available from the corresponding author upon reasonable request.

## Supplementary Materials

The following supporting information can be downloaded at: https://www.mdpi.com/article/10.3390/ijms24032338/s1.
