# Supplementary material for: sFgl2-Treg Positive Feedback Pathway Protects against Atherosclerosis
Source: Int J Mol Sci. 2023 Jan 25;24(3):2338. doi: 10.3390/ijms24032338 (PMC9916961; doi:10.3390/ijms24032338)
Supplement: Supplementary file 1 [file ijms-24-02338-s001.zip › ijms-2130056-supplementary-updated.pdf]

Sup. Figure S1

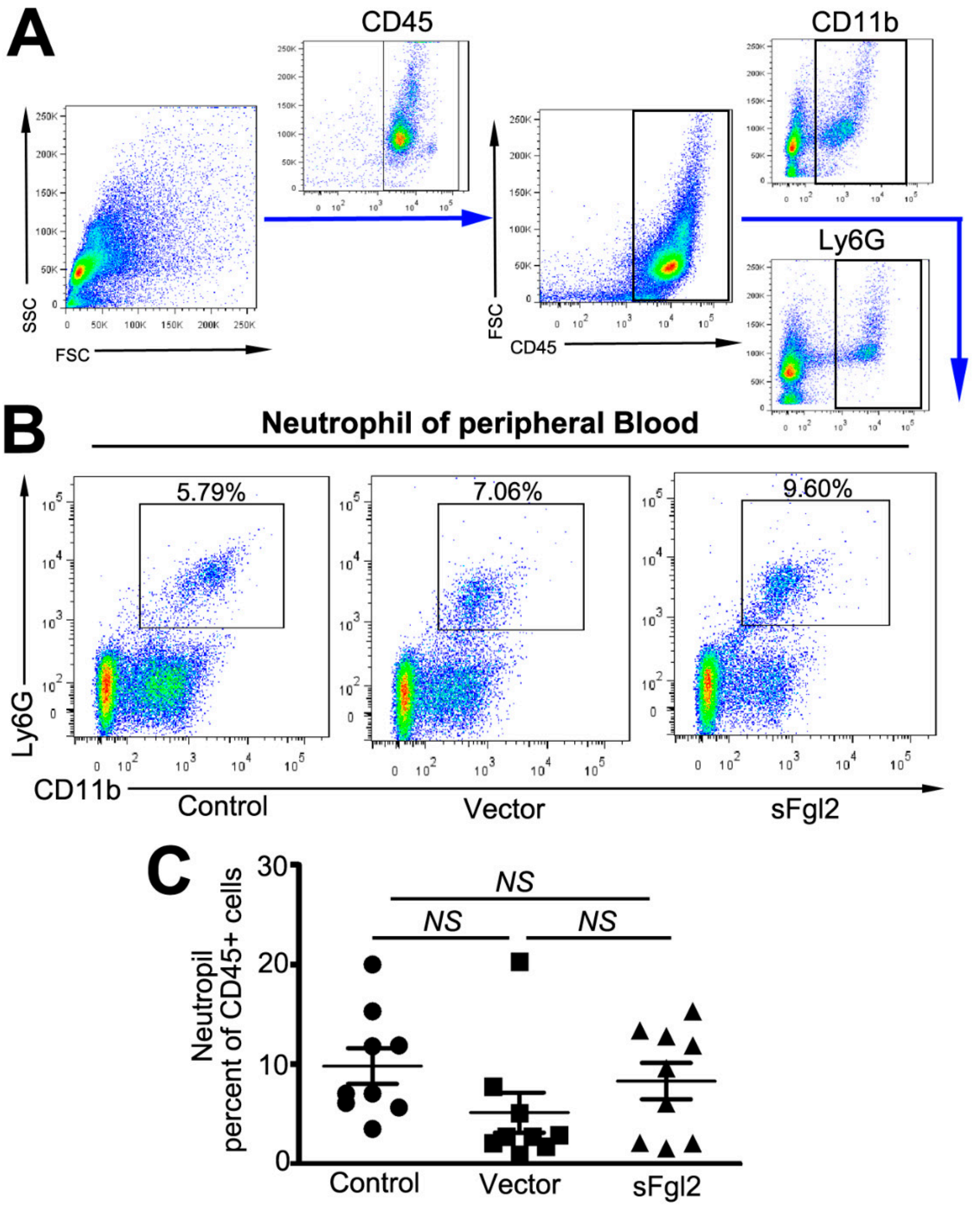

Sup. Figure S1 sFgl2-AAV exerts no effect on the Neutrophil proportion of peripheral blood.

(A-B) $CD45^+CD11b^+Ly\text{-}6G^+$ Neutrophil subset was progressively gated by FCM in the peripheral blood cells of PBS-, Vector-AAV-, and sFgl2-AAV- tail vein injected $ApoE^{-/-}$ mice. Numbers were the percentage of each subset among total monocytes. The upper plots show gating lines of CD45, Ly6G, and CD11b according to the single stained antibody. **(C)** Quantification of Neutrophil percentage in total $CD45^+$ cells. Data were represented as mean ± SEM. NS = Not Significant.

Sup.Figure S2

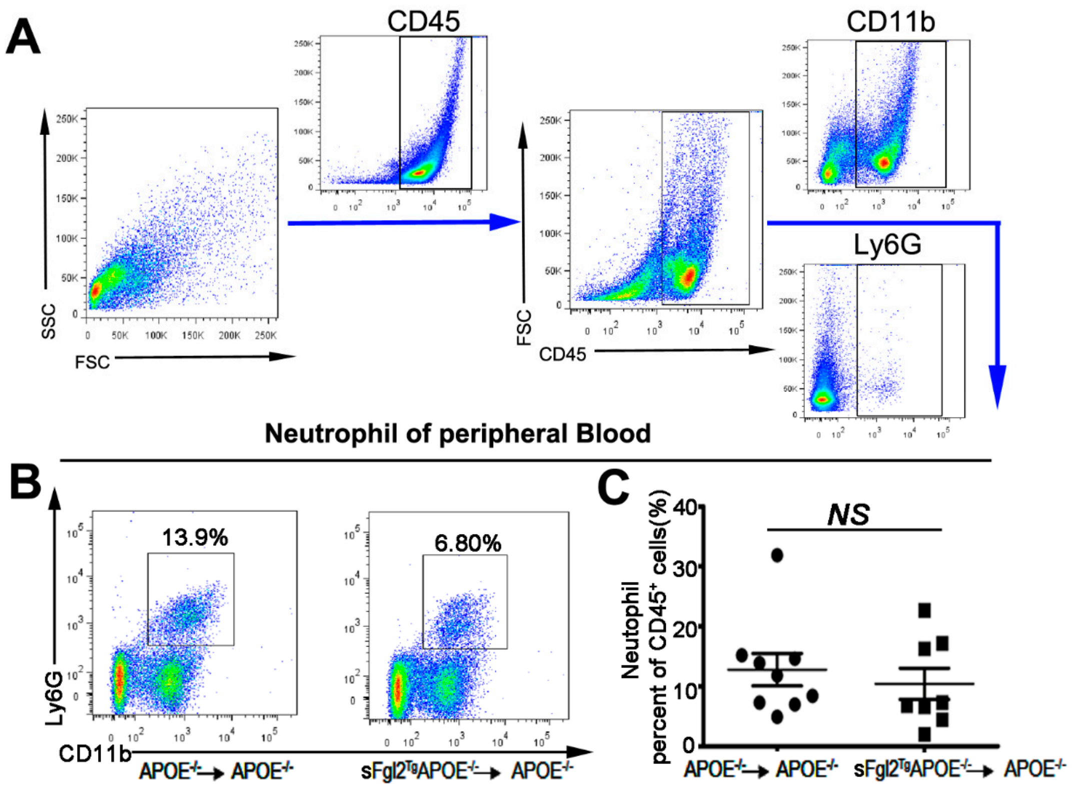

Sup. Figure S2 Bone marrow-derived sFgl2 had no influence on the number of Neutrophil in peripheral blood. **(A-B)** Peripheral blood cells were gated to determine the presence of CD45$^+$CD11b$^+$Ly-6G$^+$ Neutrophil subsets of irradiated ApoE$^{-/-}$ mice reconstituted with sFgl2^{Tg}ApoE$^{-/-}$ or ApoE$^{-/-}$ bone marrow. Numbers were the percentage of each subset among CD45$^+$ cells. The upper plots show gating lines of CD45, Ly6G, and CD11b according to the single stained antibody. **(C)** Quantification of Neutrophil percentage in CD45$^+$ cells. Data were represented as mean ± SEM. NS = Not Significant.

Sup. Table S1. No significant difference in blood lipids between groups of mice

	**AAV-Injected Mice**				**BMT Mice**		
**Lipids(mM)**	**Control**	**Vector**	**sFgl2**	***P***	**ApoE$^{-/-}$→ApoE$^{-/-}$**	**Fgl2^{Tg}ApoE$^{-/-}$→ApoE$^{-/-}$**	***P***
TC	13.77 ± 3.93	9.84 ± 1.88	8.17 ±4.85	0.316	5.15±0.78	3.47 ±0.88	0.175
TG	1.38±0.51	1.19±0.27	0.96 ± 0.23	0.697	2.08 ±0.58	0.91±0.19	0.099
HDL	5.49±3.03	2.49±0.58	3.87 ±1.06	0.534	3.01 ±0.98	2.30 ± 0.46	0.565
LDL	8.31±1.90	6.96 ±2.20	2.25±0.33	0.083	3.34 ±0.90	2.79 ±0.29	0.077

Data are means ± SEM, N= 10.

Mm, mmol/L; CHOL, total cholesterol; TG, triglyceride; HDL, high density lipoprotein cholesterol; LDL, low density lipoprotein cholesterol.
